# Development and Validation of the Mood Instability Questionnaire-Trait (MIQ-T)

**DOI:** 10.3390/medicina57080838

**Published:** 2021-08-18

**Authors:** Joohyun Yoon, Tae Hyon Ha, Sunghee Oh, Yun Seong Park, Hyun A. Ryoo, Hyeon-A Yu, Seok Joo Hong, Nayoung Cho, Chan Woo Lee, Yoonjeong Jang, Wonyun Lee, Ye Rim Kim, Kwang Ho Park, Jiung Park, Ji Yoon Park, Woojae Myung

**Affiliations:** 1 Department of Neuropsychiatry, Seoul National University Bundang Hospital, Seongnam 13619, Korea; jy3020@tc.columbia.edu (J.Y.); 35012@snubh.org (S.O.); ganbam@hanmail.net (Y.S.P.); benzcl500@naver.com (H.A.R.); hkhkh2142@naver.com (H.-A.Y.); hongsjoo0227@gmail.com (S.J.H.); purple0112@hanmail.net (N.C.); cksdnlww@gmail.com (C.W.L.); j994706@naver.com (Y.J.); md.wonyun@gmail.com (W.L.); yeriming712@gmail.com (Y.R.K.); khp93@hanmail.net (K.H.P.); jyp2128@tc.columbia.edu (J.Y.P.); 2 Department of Psychiatry, Seoul National University College of Medicine, Seoul 03080, Korea; 3 Jamsil Sky Mental Health Clinic, Seoul 05510, Korea; neuroplastic11@gmail.com

**Keywords:** mood instability, exploratory factor analysis, scale development, depression, bipolar disorder

## Abstract

*Background and objectives*: Mood instability (MI) is a stable trait associated with psychiatric disorders, yet there is a lack of tools to measure MI. The purpose of this study was to develop and validate the Mood Instability Questionnaire-Trait (MIQ-T) to evaluate MI in mood disorder patients. *Material and methods*: Items were taken from various established questionnaires to create an initial list of MIQ-T questions. Data from 309 psychiatric patients (*n* = 309; 62 major depressive disorder, 58 bipolar I disorder, and 189 bipolar II disorder) were gathered from their medical records and were utilized in an exploratory factor analysis to clarify the underlying components of MI. Then, anonymous survey data from 288 individuals from the general population were included in the analysis as a comparison group. Associations between MIQ-T and other previously validated clinical instruments for mood disorders were examined to test external validity. *Results*: The exploratory factor analysis demonstrated that the five-factor structure (Lability, Upward Tendency, Downward Tendency, Childhood Instability, and Seasonality) of 59 items was the most appropriate with clear, cohesive features. MIQ-T exhibited high internal consistency (α = 0.96) and moderate to strong correlations with other previously validated clinical instruments, which were consistent with theoretical predictions, providing evidence of criterion validity. Short forms were also created to address the high internal consistency value, which can indicate redundancy, and to increase the approachability of the measure. We found that the patients with bipolar II disorder had higher MIQ-T scores than the patients with bipolar I disorder or major depressive disorder and the comparison group. *Conclusion*: Together, these findings validate the newly developed MIQ-T as an instrument of mood instability. MIQ-T can be a potential research tool for mood disorder.

## 1. Introduction

Mood instability (MI; also called mood variability or affective instability) is a stable trait that plays an important role in psychopathology [1]. The concept of MI encompasses various areas, such as the frequency of mood changes, the lack of control over such shifts, intensity and reactivity of moods, and the related behavioral outcomes [2,3].

MI is a particularly important trait to consider for patients with bipolar disorder spectrum. Kochman et al. [4] found that children and adolescents who demonstrated MI were not only more likely to progress from major depressive disorder (MDD) to bipolar disorder (BD) but also more likely to report suicidality. Similarly, Qiu, Akiskal, Kelsoe, and Greenwood [5] reported that MI was found to be associated with increased aggression in bipolar I disorder (BD I) patients. Qiu et al. [5] additionally observed that MI was associated with an earlier age of onset and a more severe course of disorder for BD I patients, adding to the evidence that MI is considerably linked with bipolarity. In fact, MI commonly occurs as part of the prodromal symptoms in BD [6] and is known to influence patients’ prognosis even after treatment [7]. Moreover, it is possible to distinguish between the two BD subtypes depending on whether MI was experienced during a (hypo)manic or depressive episode [8]. Thus, a measure of MI can be used to predict not only the onset of BD but also its accompanying features as well as later clinical and functional outcomes. 

While research identifies MI as a noteworthy element in the field of clinical psychology and psychiatry, there lacks a specific scale that measures MI in its entirety in a systematic manner. For example, the Affective Lability Scale (ALS) is a self-report questionnaire that assesses the changeability of affect from one mood to another [9]; however, it does not encompass other key aspects of MI, such as the intensity of moods, reactivity of moods depending on external stimuli, and the resulting psychosocial and cognitive outcomes [2,3]. In addition, the Difficulties in Emotion Regulation Scale (DERS) emphasizes the cognitive aspects that lead to MI (i.e., acceptance, awareness, and clarity of emotions as well as regulation strategies) rather than focusing on the observable features and behavioral outcomes of MI [10].

Other currently available assessments of MI are scales measuring cyclothymic temperament: Temperament Evaluation of Memphis, Pisa, Paris, and San Diego Autoquestionnaire (TEMPS-A) and Cyclothymic-Hypersensitive Temperament Questionnaire (CHTQ). Cyclothymic temperament is one of the five affective temperaments identified by Akiskal and his collaborators, which is characterized by the up and down shifts in mood and energy level, as well as variability in cognition, sleep, and interpersonal attitudes [11,12]. Therefore, we can assume that individuals with a cyclothymic temperament hold the MI trait. However, the cyclothymic subscale of TEMPS-A is not an ideal measure to assess MI, as MI includes any type of affective change and intensity, while cyclothymic temperament is focused on the oscillating tendency between feeling vibrant and energetic to feeling depressed and lethargic. Similarly, the CHTQ measures one’s predisposition to cyclothymic temperament, emotional hypersensitivity, and impulsiveness; thus, it also includes some items that are related to the irritable temperament but leaves out certain aspects of MI [4,13,14].

Therefore, we propose to fill the existing gap in the literature and clinical practice by developing a tool that focuses entirely on the MI trait and measures it independently. The purpose of this study is to develop a new questionnaire that thoroughly encompasses the concept of MI and to evaluate its reliability and validity.

## 2. Materials and Methods

### 2.1. Subjects

Data from 309 psychiatric patients and 288 individuals from the general population were analyzed. Participants in the patient group had a psychiatric diagnosis and received treatment at the mood disorder clinic of Seoul National University Bundang Hospital (SNUBH) from November 2016 to July 2020. Patients were excluded from the study if they had a prior diagnosis of a clinically significant neurological disorder or a history of traumatic brain injury. For the patient group, all relevant information, including their age, gender, and DSM-5 diagnosis, were gathered from their medical records. At the time of data collection, 26.9% of patients were in the depressive state and the remaining 73.1% of patients were either in the (hypo)manic or the euthymic state. Information about (hypo)manic states of patients was not available during the retrospective chart review. Subjects from the general population, who were used as a comparison group, were recruited anonymously. Individuals were excluded from the comparison group if they self-reported a prior diagnosis of a psychiatric disorder. There were no additional screening procedures for exclusion other than the self-reported history of psychiatric diagnosis. For the present study, approval was obtained from the Institutional Review Board at SNUBH.

### 2.2. Questionnaire Development

There were several steps involved in developing this new self-report questionnaire designed to measure MI. First, we conducted an extensive literature review to identify all aspects of MI: frequency of mood changes, mood intensity, emotional reactivity, perceived ability to control moods, and related cognitive and behavioral outcomes [2,3]. Keeping this concept of MI in mind, four authors (two psychologists: JY, SO; and two psychiatrists: WM, THH) reviewed items from questionnaires that measure individuals’ temperament, personality, and behavior patterns to select the most relevant items. The list of scales and questionnaires that was initially considered can be found below in the Clinical Instruments section. After the four authors’ review, this item pool was then distributed to psychiatrists, psychologists, psychiatric nurses, and clinical researchers in the mood disorder clinic at SNUBH for their expert opinion on the items’ content validity. These 8 experts were asked to rate each item from a scale of 0 to 5, with 0 as “delete” and 5 as “strongly relevant,” and to comment specific suggestions for revision if necessary. This process ultimately resulted in a question pool of 65 items (Supplementary Table S1). Of these, 40 questions were from a modified full version of TEMPS-A (22 questions from the original full TEMPS-A and 18 additional questions that were devised by the mood clinic team at SNUBH to capture the MI trait), three from the Connor–Davidson Resilience Scale (CD-RISC; [15]), eight from the Behavioral Inhibition/Activation Scale (BIS/BAS; [16]), three from the Seasonal Pattern Assessment Questionnaire (SPAQ; [17]), six from the Personality Assessment Inventory for Borderline Features Scale (PAI-BOR; [18]), and five from the Wender Utah Rating Scale-25 (WURS-25; [19]).

### 2.3. Clinical Instruments

Modified TEMPS-A and PAI-BOR were used to evaluate temperament and personality. TEMPS-A measures an individual’s affective temperaments, which are important to consider in terms of emotional reactivity and psychosocial functioning [20]. Similarly, PAI-BOR is a scale within the PAI that focuses on mood lability as well as unstable self-identity and interpersonal relationships [18,21]. Childhood trauma, which is known to be associated with affective instability and the development of mood disorder later in life [22,23], was measured using the Childhood Trauma Questionnaire, Short Form (CTQ-SF) [24]. Four questionnaires were used to assess behavioral patterns: Interpersonal Sensitivity Measure (IPSM), CD-RISC, BIS/BAS, and SPAQ. IPSM measures one’s tendency to be hypersensitive to interpersonal relationships and rejection [25]. CD-RISC, on the other hand, assesses an individual’s ability to adapt to change and cope with stress [15]. BIS/BAS measures how an individual’s motivation system regulates his or her action [16]. As individuals’ mood and behavior can be heavily affected by seasonal changes, SPAQ was used to assess how the subjects felt in terms of emotional variability, energy, and functioning across seasons [17]. Lastly, the presence of clinical symptoms was assessed using three questionnaires: WURS-25, Beck Anxiety Inventory (BAI), and Zung Self-Rating Depression Scale (SDS). WURS-25 retrospectively measures childhood ADHD symptoms. BAI gathers data regarding anxiety symptoms felt in the last seven days [26]. Similarly, SDS asks about the presence and severity of depression symptoms, including mood, cognition, and psychosocial outcomes, over the past two weeks [27]. Of these instruments, TEMPS-A, PAI-BOR, CTQ-SF, IPSM, CD-RISC, BIS/BAS, BAI, and SDS were also used for external validation.

### 2.4. Statistical Analysis

The initial question pool of 65 items was subjected to an exploratory factor analysis with the data collected from the patient group. Exploratory factor analysis was conducted using the method of principal component analysis with direct oblimin rotation (delta = 0). We used a variety of methods to determine the most appropriate number of factors that best explained the structure of the scale. We ensured that each factor is distinct from one another, and that they all correspond to the MI trait. Items that loaded onto a single factor with a factor loading of 0.40 or above were retained. Items that loaded onto several factors with a loading of 0.40 or greater (i.e., items with cross-loadings) were removed. 

After developing the Mood Instability Questionnaire-Trait (MIQ-T), several analyses were conducted to test its reliability and validity. Cronbach’s alpha (α) was used to verify the internal consistency of the factors and the entire scale [28]. We computed descriptive statistics for demographic and clinical variables, and we calculated age and gender differences in the sample groups using independent samples t-test and chi-square test. Short forms were also created to increase the ease of administration and lower participant burden. First, we conducted a new exploratory factor analysis of the 59 MIQ-T items using a principal component analysis with direct oblimin rotation (delta = 0). As our purpose was to select items that strongly represent MI, we used two criteria to evaluate items. Items that showed factor loadings greater than 0.50 on its original factor with low loadings (<0.30) on other factors were retained. Then, we examined the item-total correlations for each factor and items that had a correlation coefficient greater than 0.50 were kept, producing a 30-item short form (MIQ-T SF-30). We wanted to reduce this short form further into a 15-item version, so another exploratory factor analysis via principal component analysis with direct oblimin rotation (delta = 0) was conducted on the remaining 30 items. Items with the highest factor loadings for each factor were retained, producing MIQ-T SF-15. To validate the short forms, internal consistency was tested, and multiple linear regression analyses were performed. To determine whether MIQ-T has external validity, we compared MIQ-T with pre-existing, validated instruments using a Pearson correlation analysis while controlling for age and gender. Multiple regression analyses were performed to analyze the capability of MIQ-T in differentiating between diagnosis groups, and between the patient group and the comparison group. All statistical analyses were two-tailed, with the statistical significance level set at <0.05. All statistical analyses were conducted on IBM SPSS version 27.0.

## 3. Results

### 3.1. Clinical and Demographic Characteristics

The clinical and demographic characteristics are shown in Table 1. Participants’ age ranged from 15 to 66, with a mean of 34.09 (standard deviation (SD) = 10.87). There were 202 male and 395 female subjects in total, with 93 males and 216 females in the patient group and 109 males and 179 females in the comparison group.

### 3.2. Exploratory Factor Analysis

Before conducting and interpreting the exploratory factor analysis, we first determined the fit and suitability of our sample data. Two indicators were examined to this effect: the Kaiser–Meyer–Olkin (KMO) measure of sampling adequacy and the Bartlett’s test of sphericity. Both were at acceptable standards with a KMO value of 0.93 and the significance level of Bartlett’s test of sphericity at *p* < 0.001. We submitted the primary list of 65 items to a principal component analysis with direct oblimin rotation, as we expected the factors to be correlated. Various methods including parallel analysis, Kaiser’s criterion, 50% variance cutoff, and scree plot elbow rule were attempted to ensure an appropriate factor structure with a clear loading pattern. The five-factor model, which included a total of 59 items and accounted for 49.70% of the variance, was chosen for its conceptual clarity (see Table 2 for the English version of MIQ-T factor structure and Supplementary Table S2 for the Korean version). 

The first factor, which contained 26 items, was named Lability and measures an individual’s shifts in mood, energy, cognition, and social behavior. The second factor, composed of six items, was labeled Upward Tendency, as it focuses on the propensity for an individual to feel positive and energetic. The third factor of 18 items was named Downward Tendency and describes an individual’s likelihood to feel depressed, anxious, and irritable. It also contains some items regarding the instability of one’s emotions and reactivity to stress. The fourth factor, loaded on by 6 items, was labeled Childhood Instability, although it contained five items relating to childhood and one item regarding the quality of interpersonal relationships. We believe this latter item was loaded under this factor as the pattern of attachment or the method of emotional processing developed earlier in childhood may impact the current patterns of interpersonal relationships. The final factor containing three items was named Seasonality, as it measures the variations in an individual’s mood, energy, and behavior that may occur with seasonal changes. Scoring was adjusted for some items to standardize the minimum score into 0. The maximum score for MIQ-T was 188.

### 3.3. Reliability 

The reliability of MIQ-T was assessed with Cronbach’s coefficient α to measure internal consistency. The Cronbach’s alpha value for the full questionnaire was 0.96, and the alpha values for the five factors were 0.95, 0.78, 0.93, 0.87, and 0.82, respectively. All alpha values were higher than the most commonly accepted standard, which is 0.70 [29].

## 4. Concurrent Validity

### 4.1. Correlation with TEMPS-A

To ensure that our questionnaire encompasses the features within the concept of MI, partial correlation of MIQ-T with the cyclothymic subscale of TEMPS-A short version was conducted. There was a strong, significant correlation between the MIQ-T score and the cyclothymic score in TEMPS-A short version for both the patient and the comparison group (r = 0.79, *p* < 0.01 and r = 0.77, *p* < 0.01, respectively). The MIQ-T score also demonstrated a moderate to strong correlation to all other subscales of TEMPS-A, except for the hyperthymic temperament (Table 3).

### 4.2. Correlation with Other Scales

MIQ-T was further correlated with other scales relating to one’s clinical symptoms, personal history, and behavioral patterns (Table 3). Firstly, MIQ-T showed strong correlations with BAI and SDS, which are scales relating to anxiety and depression symptoms respectively: for BAI, r = 0.62, *p* < 0.01 for patient group and r = 0.78, *p* < 0.01 for comparison group; for SDS, r = 0.63, *p* < 0.01 for patient group and r = 0.74, *p* < 0.01 for comparison group. Secondly, the correlations for both populations were found to be moderate when using the CTQ total score (r = 0.37, *p* < 0.01 for patient group and r = 0.54, *p* < 0.01 for comparison group). Within the CTQ subscales, emotional abuse exhibited the highest correlation coefficient (r = 0.40, *p* < 0.01 for patient group; r = 0.51, *p* < 0.01 for comparison group). Finally, MIQ-T score was also compared with clinical scales for behavioral patterns. A positive correlation was found between MIQ-T and IPSM (r = 0.67, *p* < 0.01 for patient group and r = 0.61, *p* < 0.01 for comparison group), while there was a moderate inverse relationship between MIQ-T and CD-RISC (r = −0.40, *p* < 0.01 for patient group and r = −0.43, *p* < 0.01 for comparison group). With BIS/BAS scales, BIS (r = 0.63, *p* < 0.01 for patient group and r = 0.56, *p* < 0.01 for comparison group) exhibited a stronger correlation with MIQ-T than BAS (r = 0.32, *p* < 0.01 for patient group and r = 0.22, *p* < 0.01 for comparison group) did. With regard to PAI-BOR, it showed the strongest correlation with MIQ-T than any other scales listed above (r = 0.79, *p* < 0.01 for patient group; r = 0.85, *p* < 0.01 for comparison group). 

### 4.3. Comparison of MIQ-T Scores According to Diagnosis

Average MIQ-T scores were significantly different (ß = 0.45, *p* < 0.001) between the comparison group and the bipolar II disorder (BD II) group (mean = 67.47, SD = 27.84 for comparison group vs. mean = 98.69, SD = 26.58 for BD II, Table 4). The mean scores were also significantly different (ß = 0.15, *p* < 0.01) between the comparison group and the major depressive disorder (MDD) group (mean = 67.47, SD = 27.84 for comparison group vs. mean = 77.32, SD = 28.70 for MDD, Table 4). There were significant differences in average MIQ-T scores between the different mood disorder diagnoses as well: both the MDD group and BD I group were significantly different from the BD II group (mean = 77.32, SD = 28.70 for MDD vs. mean = 98.69, SD = 26.58 for BD II, ß = 0.20, *p* < 0.01; mean = 71.36, SD = 34.72 for BD I vs. mean = 98.69, SD = 26.58 for BD II, ß = 0.35, *p* < 0.001, Table 4).

There were also significant differences in average MIQ-T factor scores between groups. For Factor 1 (Lability) and Factor 3 (Downward Tendency), all between-group differences were found to be significant, with the exception of the mean scores between the comparison group and BD I group. For Factor 2 (Upward Tendency), average scores were significantly different between the comparison group and MDD group, and between the MDD group and BD II group. For Factor 4 (Childhood Instability), average scores were significantly different only between the comparison group and BD II group. There were no significant differences found between all groups for Factor 5 (Seasonality). Details of the comparison of average MIQ-T total scores and factor scores can be found in Table 4.

### 4.4. Development of MIQ-T Short-Form

MIQ-T demonstrated a high level of internal consistency (α = 0.96 for full questionnaire). Some studies have suggested that alpha values greater than 0.90 may imply redundancy [29,30]. Short forms of MIQ-T were created to address this issue and to increase the approachability of the measure to be used in research and clinical settings. 

A new exploratory factor analysis was performed on the 59 items of MIQ-T via principal component analysis with direct oblimin rotation (delta = 0), based on the patient group data. The KMO value was 0.93 and Bartlett’s test was significant (*p* < 0.001), demonstrating the adequacy of the sample. Items with factor loadings greater than 0.50 on its intended factor and low loadings (less than 0.30) on other factors were retained. In addition, item-total correlations were computed for each factor with the remaining items, and only items that had a correlation coefficient greater than 0.50 were kept, resulting in a 30-item short form (MIQ-T SF-30). It should be noted that the fifth factor, Seasonality, was not maintained in MIQ-T SF-30 as original items in this factor did not show significant factor loadings and only displayed cross-loadings. 

In order to develop a shorter version to further decrease administration time, another exploratory factor analysis was conducted on the remaining 30 items, in which 15 items with the highest factor loadings were retained. We believed 15 would be a sufficient number of questions to ensure thorough assessment of patients’ MI trait while lowering the respondent burden. Six items were chosen from the first factor, Lability, as it included the greatest number of items in the full version of MIQ-T. Three items each were chosen from the rest of the factors. Items retained in SF-30 and SF-15 and the corresponding factor loadings can be found in Supplementary Tables S3 and S4.

The internal consistency values were high for both short forms (MIQ-T SF-30: α = 0.94, MIQ-T SF-15: α = 0.89). Linear regression analysis showed that MIQ-T SF-30, with age and sex as covariates, explained 96% of the variance of the MIQ-T full questionnaire (R^2^ = 0.96). MIQ-T SF-30 had a strong association to the MIQ-T full questionnaire (ß = 0.98, *p* < 0.001). Similarly, MIQ-T SF-15 demonstrated great predicting power, explaining 89% of the variance of the MIQ-T full questionnaire (R^2^ = 0.89), and SF-15 was significantly associated with MIQ-T full questionnaire (ß = 0.94, *p* < 0.001).

## 5. Discussion

The purpose of this study was to develop and evaluate the validity of MIQ-T, a self-report instrument designed to measure the MI trait. By using exploratory factor analysis, we identified 59 items that loaded onto five factors capturing the comprehensive concept of MI. Results indicated that MIQ-T is internally consistent, with a high Cronbach’s alpha value of 0.96 for the full questionnaire. When compared with previously validated clinical instruments, MIQ-T demonstrated correlations that are consistent with theoretical predictions. This study also observed that MIQ-T can successfully differentiate between those with mood disorders and the nonclinical population. Moreover, we found that MIQ-T may be used to distinguish between mood disorders.

MIQ-T is a 59-item self-report questionnaire with five factors: Lability (26 items), Upward Tendency (6 items), Downward Tendency (18 items), Childhood Instability (6 items), and Seasonality (3 items). The factor structure is consistent with the literature, which show that MI includes aspects of valence, sudden and frequent shifts, intensity, and reactivity to external environment [2,3]. Lability incorporated the abrupt and intense oscillations of mood, energy, and cognition, whereas Upward Tendency and Downward Tendency included different affective valences. These three factors also contained the feature of mood reactivity. The factor of Childhood Instability consisted mostly of items taken from WURS-25. Childhood Instability was included as a factor for MIQ-T because the relationship between childhood attention deficit hyperactivity disorder and adult mood disorders has been established [31]. Joo, Lee, Choi, Kim, Song, Bang, Ahn, and Kim [31] claimed that patients with mood disorders exhibited higher scores on impulsivity, inattention, and mood instability factors within the WURS-25 scale. Lastly, Shin et al. [32] pointed out that individuals with bipolar disorder reported greater seasonality than those with depression and the control sample, suggesting that MI is related to seasonality. Therefore, the five-factor structure of MIQ-T seem to accurately represent the essence of MI.

Concurrent validity of the questionnaire was found in correlation analyses with relevant scales. All correlations were consistent in both the patient group and the comparison group. Firstly, MIQ-T was strongly correlated with the cyclothymic subscale of TEMPS-A short version. This is in alignment with previous research which demonstrated that BD patients, especially BD II patients, exhibit high levels of cyclothymic temperament (i.e., instability in mood, cognition, and behavior) even in the absence of mood episodes [33,34]. Nowakowska et al. [33] demonstrated that the higher cyclothymic temperament scores of euthymic BD patients could be used to differentiate them from the healthy subjects [33,34]. In addition, research has indicated that the definition of MI not only includes the shifting of moods from one pole to another, but also the tendency to progress in a specific direction in an emotional situation. With regard to such context, MIQ-T is also significantly correlated with other subscales of the TEMPS-A short version, with the exception of the hyperthymic subscale that is known to have a protective effect on mental disorders [35]. Moreover, Rozsa et al. [36] previously demonstrated this divide between the TEMPS-A subscales. A principal component analysis of TEMPS-A revealed a two-factor solution in which Factor 1 consisted of the cyclothymic, depressive, irritable, and anxious subscales while Factor 2 consisted of the hyperthymic subscale. Thus, it is viable to interpret that these four affective temperaments represent a single dimension, which may be linked with the concept of MI. 

The strongest correlation between MIQ-T and a pre-existing scale was demonstrated by PAI-BOR, despite having taken only a few items from the PAI-BOR scale when developing MIQ-T. This is consistent with the literature on MI, as MI is known to be a characteristic feature of borderline personality disorder [37,38,39] and can be used as one of the discriminant dimensions that differentiates those diagnosed with borderline personality disorder from a community sample and those with other psychiatric disorders [40,41].

Since individuals who possess the MI trait are thought to be sensitive to the external environment, we expected MIQ-T to be at least moderately correlated with IPSM and CD-RISC. In line with our expectations, our calculation demonstrated a strong correlation of MIQ-T with IPSM and a moderate, inverse relationship with CD-RISC. 

Although there was only a moderate correlation between MIQ-T and CTQ, the subscale that had the strongest correlation was emotional abuse, reflecting the dose–effect association between emotional abuse and bipolar disorder [42] and the detrimental effect of childhood emotional abuse on emotional development [43]. 

MIQ-T also exhibited significant correlations with BAI and SDS, which are used to assess symptom severity. This finding may add to the evidence that MIQ-T is a valid measure, as MI is known to be associated with depression and anxiety disorders [44]. However, as BAI and SDS measure the current state of patients, correlations with these two scales may suggest that MIQ-T, although developed to measure one’s MI trait, may be predicting the state of a person rather than one’s existing traits.

In terms of using MIQ-T in clinical settings, our results suggest that MIQ-T can be helpful for differential diagnosis. Multiple linear regression analyses have found between-group differences in MIQ-T factor scores for diagnosis groups, with the exception of Factor 5 (Seasonality). For Factor 1 (Lability) and Factor 3 (Downward Tendency), the general pattern seemed to be that the comparison group and BD I group have similar scores, with the MDD group scoring slightly higher than these two groups, and the BD II group scoring the highest. There were between-group differences for all group pairs with the exception of comparison group vs. BD I group. For Factor 1 (Lability), more significant differences in means were demonstrated between the comparison group and BD II group (ß = 0.42, *p* < 0.001), between the BD I group and BD II group (ß = 0.34, *p* < 0.001), and between the MDD group and BD II group (ß = 0.18, *p* < 0.01). Thus, MIQ-T Factor 1 (Lability) can be seen as what differentiates BD II from the general population and from other psychiatric diagnoses. In comparison, for Factor 3 (Downward Tendency), the more significant differences in means were shown between the comparison group and MDD group (ß = 0.27, *p* < 0.001), between the comparison group and BD II group (ß = 0.52, *p* < 0.001), and between the BD I group and BD II group (ß = 0.39, *p* < 0.001). Therefore, Factor 3 (Downward Tendency) can be seen as the most important aspect of MI in terms of recognizing the clinical population from the nonclinical population and distinguishing between the two BD subtypes. 

On the other hand, the patterns for mean MIQ-T Factor 2 (Upward Tendency) and Factor 4 (Childhood Lability) scores were slightly different: for Factor 2 scores, there was an increasing pattern from MDD to the comparison group to BD I to BD II, while for Factor 4, the mean scores increased from the comparison group to MDD to BD I to BD II. For Factor 2, average scores were significantly different for the comparison group vs. MDD group (ß = −0.12, *p* < 0.05) and MDD group vs. BD II group (ß = 0.14, *p* < 0.05). For Factor 4, average scores were significantly different for the comparison group vs. BD II group (ß = 0.22, *p* < 0.001). Results from multiple linear regression analyses indicate that MIQ-T will be useful in identifying those who are healthy from those with mood disorders as well as in distinguishing BD II from the other two mood diagnoses of MDD and BD I.

Overall, the BD II group scored higher in MIQ-T, while the BD I group appeared similar to the comparison group. This result can be explained by previous research that points out the distinct temperament profile of BD II patients [45]. Akiskal, Kilzieh, Maser, Clayton, Schettler, Traci Shea, Endicott, Scheftner, Hirschfeld, and Keller [45] explains that BD II patients scored higher on temperamental dimensions of mood lability, energy–assertiveness, and sensitivity–brooding, while BD I patients scored similarly to controls in affective stability. Therefore, BD II patients seem to have more distinguishable personality traits when compared to the general population than those with other mood disorders (e.g., MDD and BD I). 

There were two short forms that were developed to make MIQ-T more approachable and user-friendly: MIQ-T SF-30 and MIQ-T SF-15. Both short forms showed high Cronbach’s alpha value (α = 0.94, α = 0.89, respectively), demonstrating good internal consistency. The results of linear regression analyses indicated that SF-30 explained 96% of the variance of the MIQ-T full questionnaire, whereas SF-15 explained 89% of the variance of the MIQ-T full. Therefore, both SF-30 and SF-15 seem to be reliable indicators for MIQ-T full, allowing them to be dependably used as instruments of MI in academic and clinical research.

Several limitations exist in this study. First of all, a major limitation of the study is that the absence of psychiatric diagnoses in the comparison group was not confirmed via interview but was based on self-report. Although this would not have impacted the factor analysis results, since all exploratory factor analyses were conducted with the data collected from patient group, this may have adversely impacted the association tests between the patient group and comparison group. Secondly, not all information about the mood state of patients was available during the retrospective chart review. While 26.9% of patients were in the depressive state at the time of data collection, the percentage of patients in the (hypo)manic state could not be calculated, as the information was not available. Mood states of patients are important to consider as they may have influenced MIQ-T scores. In order to effectively compare healthy controls and psychiatric patients, future studies should not only confirm the absence of psychiatric diagnoses in the control group but also confirm the mood states of patients at the time of data collection. Another limitation regarding the sample is that the original development and data collection were conducted in Korean and involved only Korean participants. There may be different presentations of MI across societies and cultures, which may impact the reliability and validity of this scale. A further validation study must be conducted in other languages and with a culturally diverse sample. Another shortcoming is that the sample demographic ratios were not proportionate. Although we attempted to form comparable samples for the comparison group and patient group, it was challenging to pursue this, as it was partly a retrospective study in which the data from patients were collected via chart review, while the data from comparison group were obtained via an anonymous survey. Moreover, the sample was not equal between different clinical groups, with a much larger sample size of BD II patients than that of MDD and BD I. We also had a larger proportion of older participants with a diagnosis of MDD compared to the two BD groups, which impacts the reliability and validity of using MIQ-T to distinguish between diagnoses. Follow-up studies could address these limitations involving the sample by constructing culturally diverse, balanced sample groups. Furthermore, since we selected questions from various scales to develop a new questionnaire, the answer choices were not uniform across all items. For convenience and approachability in future use, there is a need to determine whether this impacts the validity of the scale and to enforce a uniform mode of scoring. The sixth limitation of the present study is that specifiers of DSM-5 diagnoses were not considered. For example, an individual who is diagnosed as MDD with mixed features may present differently in terms of MI when compared with an individual who is diagnosed as MDD with melancholic features. We believe this could be part of an explanation used to clarify why MDD patients demonstrated a higher mean MIQ-T total score than BD I patients in this study. Some of these MDD patients in our sample may have been suffering from mixed features depression or agitated depression, which is more likely to cause mood lability and irritability than other types of MDD [46]. To address this limitation, future studies should include specifiers of psychiatric diagnoses in their statistical analyses. Lastly, although MIQ-T demonstrated expected correlations with other validated instruments that are currently in use with mood disorder patients, we recognize that this may not be the best assessment of the reliability and validity of MIQ-T, as these instruments have been developed to measure different clinical constructs. Follow-up research is needed to further evaluate and confirm the content validity of MIQ-T.

## 6. Conclusions

This study describes the development and initial validation of a new questionnaire, named MIQ-T, that encompasses all aspects of MI. Our results indicate that the five-factor solution of 59 items demonstrates conceptual clarity, internal consistency, and concurrent validity. In terms of clinical utility, the researchers of this study believe that MIQ-T can be useful in differentiating BD II from other mood disorders and from the general population. Future studies could further utilize the full MIQ-T questionnaire as well as the short forms to aid their measurement of MI in clinical and academic research. This could include investigating the relationship of MI to medication response, clinical course, personality dimensions, and cognitive and interpersonal factors. As previously mentioned, whether or not patients with the same psychiatric diagnoses, but with different specifiers, exhibit varying MIQ-T scores could also be explored. Such research studies could illuminate the currently elusive dimensions within mood disorders, revealing patterns that could not be observed with the DSM-5 diagnosis classification structure.

## Figures and Tables

**Table 1:** Clinical and demographic characteristics.

	Total (*n* = 597)	Patient Group (*n* = 309)	Comparison Group (*n* = 288)	*p* Value
*Age*				
Mean	34.09	32.61	35.67	
SD	10.87	11.49	9.94	0.001
*Gender*				
Male	202 (33.8%)	93 (30.1%)	109 (37.8%)	
Female	395 (66.1%)	216 (69.9%)	179 (62.2%)	0.046
*Diagnosis*				
Major Depressive Disorder	-	62 (20.1%)	-	-
Bipolar I Disorder	-	58 (18.8%)	-	-
Bipolar II Disorder	-	189 (61.2%)	-	-

**Table 2:** Exploratory factor analysis with direct oblimin rotation and the factor structure of MIQ-T in patients (*n* = 309) (items in English).

Item (English)	Factor 1	Factor 2	Factor 3	Factor 4	Factor 5
*Factor #1—Lability*					
When I have a lot of free time, my moods become unstable.	**0.759**	0.123	−0.047	−0.030	−0.024
When I have a lot of free time, I feel depressed.	**0.722**	−0.003	0.026	−0.076	0.048
When I have a lot of free time, I feel irritable.	**0.695**	−0.103	0.039	−0.113	0.125
I constantly switch from being lively and sluggish.	**0.669**	0.164	0.156	0.047	−0.180
When something good happens, I feel irritable.	**0.666**	−0.160	−0.053	0.015	0.345
I get sudden shifts in mood and energy.	**0.641**	0.204	0.165	0.048	−0.249
When I have a lot of free time, I feel anxious.	**0.634**	−0.173	0.145	−0.014	0.177
When something good happens, I feel despondent.	**0.630**	−0.289	−0.031	−0.066	0.320
The way I see things is sometimes vivid, but at other times lifeless.	**0.628**	0.181	0.121	0.010	−0.088
My ability to think varies greatly from sharp to dull for no apparent reason.	**0.611**	0.039	0.138	0.042	−0.092
My need for sleep varies a lot from just a few hours to more than 9 hours.	**0.593**	0.108	−0.016	0.081	−0.086
When something good happens, my moods becomes unstable.	**0.590**	−0.013	0.020	0.137	0.184
My mood often changes for no reason.	**0.588**	0.059	0.230	0.042	−0.156
I can really like someone a lot and then completely lose my interest in them.	**0.582**	0.141	0.035	0.045	−0.047
My moods and energy are either high or low, rarely in between.	**0.570**	0.054	0.149	0.159	−0.236
I go back and forth between being outgoing and being withdrawn from others.	**0.540**	0.147	0.063	0.128	−0.065
When something good happens, I feel anxious.	**0.519**	−0.365	0.172	0.109	0.335
My attitudes about myself changes a lot.	**0.518**	0.268	0.094	0.149	−0.116
I sometimes go to bed feeling great and wake up in the morning feeling like life is not worth living.	**0.503**	−0.266	0.291	0.121	−0.148
My mood shifts very suddenly.	**0.489**	0.232	0.121	0.202	−0.290
Sometimes, I feel terribly empty inside.	**0.484**	−0.022	0.270	0.201	−0.081
I daydream a great deal about things that other people consider impossible to achieve.	**0.479**	0.158	−0.025	0.159	0.081
I go back and forth between feeling overconfident and feeling unsure of myself.	**0.448**	0.121	0.167	0.203	−0.123
I often feel tired for no reason.	**0.448**	−0.081	0.341	0.115	−0.022
When I am busy, I feel more energetic.	**0.413**	0.317	−0.372	−0.058	−0.033
I am the kind of person who can be sad and happy at the same time.	**0.402**	0.164	−0.062	0.057	0.042

*Factor #2—Upward Tendency*					
When I get something I want, I feel excited and energized.	−0.041	**0.742**	0.073	0.023	0.058
When good things happen to me, it affects me strongly.	0.037	**0.696**	0.112	−0.034	0.068
When I see an opportunity for something I like, I get excited right away.	−0.043	**0.669**	0.085	0.204	0.140
It would excite me to win a contest.	0.019	**0.575**	−0.031	0.052	0.098
I often act on the spur of the moment.	0.135	**0.556**	0.001	0.257	0.026
When something good happens, I feel intensely euphoric.	0.207	**0.530**	0.040	0.051	−0.012

*Factor #3—Downward Tendency*					
When I experience something difficult, I feel anxious.	0.106	0.102	**0.671**	−0.006	0.011
When I experience something difficult, I get depressed easily.	0.157	0.044	**0.670**	0.039	−0.043
Criticism or scolding hurts me quite a bit.	−0.096	0.360	**0.616**	−0.138	0.062
When I am busy, I feel anxious.	0.044	−0.085	**0.608**	0.064	0.352
I feel pretty worried or upset when I think or know somebody is angry at me.	−0.052	0.374	**0.593**	−0.209	0.051
When I experience something difficult, I am more likely to get irritable.	0.047	0.103	**0.581**	0.174	0.052
I am able to adapt to change. *	0.060	−0.138	**0.557**	−0.044	−0.179
I can handle unpleasant and painful feelings, such as sadness, fear, and anger. *	0.155	−0.041	**0.550**	0.049	−0.147
I get stressed by minor changes in my daily life.	0.336	−0.020	**0.534**	−0.007	0.031
When I experience something difficult, my moods become unstable.	0.328	0.159	**0.532**	0.005	−0.018
When I am busy, I feel depressed.	0.035	−0.129	**0.526**	0.024	0.389
Even when I am under pressure, I can focus and think clearly. *	0.101	−0.078	**0.524**	0.199	−0.234
When I am busy, I easily get irritable.	0.032	0.037	**0.515**	0.184	0.303
I am an insecure person.	0.397	−0.130	**0.491**	0.154	−0.123
I am told that I often get pessimistic about things and forget previous happy times.	0.220	−0.173	**0.485**	0.247	−0.025
My mood is very steady. *	0.320	−0.110	**0.448**	0.105	−0.258
If I think something unpleasant is going to happen, I usually get pretty "worked up".	0.112	0.378	**0.435**	−0.052	0.130
When I am busy, my moods become unstable.	0.231	0.096	**0.419**	0.105	0.312

*Factor #4—Childhood Instability*					
As a child, I was hot- or short-tempered, with a low boiling point.	−0.134	0.057	−0.036	**0.862**	0.071
As a child, I acted without thinking and I was impulsive.	−0.099	0.053	−0.088	**0.835**	0.014
As a child, I was moody with ups and downs.	0.035	0.021	−0.060	**0.822**	0.121
As a child, I lost control of myself.	0.006	−0.028	−0.081	**0.765**	0.109
As a child, I had temper outbursts and tantrums.	−0.025	−0.005	−0.011	**0.705**	−0.010
My relationships have been stormy.	0.099	−0.091	0.353	**0.418**	−0.160

*Factor #5—Seasonality*					
How much do you experience seasonal variation in energy?	0.023	0.236	−0.025	0.151	**0.555**
How much do you experience seasonal variation in mood?	0.061	0.285	0.019	0.122	**0.523**
How much do you experience seasonal variation in social activity?	−0.015	0.215	0.071	0.136	**0.522**

*Notes:* Factor loadings with absolute value greater than 0.40 are shown in bold. * indicates items that were reverse-coded. Extraction Method: Principal Component Analysis. Extraction Method: Oblimin with Kaiser normalization.

**Table 3:** Partial correlation of MIQ-T total score with other clinical scales, controlling for the variables of age and gender.

	Patient Group		Control Group	
	Partial Correlation	*p*-Value	Partial Correlation	*p*-Value
*Temperament and Personality* *				
TEMPS-A				
TEMPS-A (Short) Cyclothymic	**0.79**	<0.001	**0.77**	<0.001
TEMPS-A (Short) Depressive	**0.56**	<0.001	**0.66**	<0.001
TEMPS-A (Short) Irritable	**0.45**	<0.001	**0.56**	<0.001
TEMPS-A (Short) Hyperthymic	−0.03	0.619	−0.05	0.365
TEMPS-A (Short) Anxious	**0.42**	<0.001	**0.43**	<0.001
PAI-BOR	**0.79**	<0.001	**0.85**	<0.001
*Childhood Trauma*				
CTQ Total	**0.37**	<0.001	**0.54**	<0.001
CTQ Emotional Abuse	**0.40**	<0.001	**0.51**	<0.001
CTQ Physical Abuse	**0.28**	<0.001	**0.41**	<0.001
CTQ Sexual Abuse	**0.15**	0.010	**0.38**	<0.001
CTQ Emotional Neglect	**0.26**	<0.001	**0.39**	<0.001
CTQ Physical Neglect	**0.24**	<0.001	**0.40**	<0.001
*Behavioral Patterns*				
IPSM	**0.67**	<0.001	**0.61**	<0.001
CD-RISC	**−0.40**	<0.001	**−0.43**	<0.001
BIS	**0.63**	<0.001	**0.56**	<0.001
BAS	**0.32**	<0.001	**0.22**	<0.001
*Clinical Symptoms*				
BAI	**0.62**	<0.001	**0.78**	<0.001
SDS	**0.63**	<0.001	**0.74**	<0.001

*Notes:* Significant correlations are shown in bold. * The associations were tested in the sub-population due to missing data (*n* = 268).

**Table 4:** Comparison of MIQ-T scores according to diagnosis.

	Comparison Group	Major Depressive Disorder (MDD)	Bipolar I Disorder (BD I)	Bipolar II Disorder (BD II)	Significant Associations
Factor 1 (Lability)	25.89 (14.96)	30.44 (16.14)	26.62 (16.67)	42.04 (15.83)	CG vs. MDD *; CG vs. BD II ***; MDD vs. BD I *; MDD vs. BD II **; BD I vs. BD II ***
Factor 2 (Upward Tendency)	10.50 (3.05)	9.24 (4.07)	10.34 (3.95)	10.87 (3.79)	CG vs. MDD *; MDD vs. BD II *
Factor 3 (Downward Tendency)	23.86 (9.01)	30.42 (9.34)	26.19 (11.25)	35.37 (8.06)	CG vs. MDD ***; CG vs. BD II ***; MDD vs. BD I **; MDD vs. BD II *; BD I vs. BD II ***
Factor 4 (Childhood Instability)	3.92 (4.69)	4.18 (4.82)	4.84 (5.80)	6.68 (5.39)	CG vs. BD II ***
Factor 5 (Seasonality)	3.31 (2.48)	3.05 (2.91)	3.36 (3.16)	3.72 (3.20)	None
Factor Sum/ Total	67.47 (27.84)	77.32 (28.70)	71.36 (34.72)	98.69 (26.58)	CG vs. MDD **; CG vs. BP II ***; MDD vs. BD II **; BD I vs. BD II ***

*Notes:* Mean (SD), Associations were test by multiple regression analysis after controlling for age and sex. * *p* < 0.05, ** *p* < 0.01, *** *p* < 0.001.

## Data Availability

The data presented in this study are available within the article and the Supplementary Materials.

## Supplementary Materials

The following are available online at https://www.mdpi.com/article/10.3390/medicina57080838/s1, Supplementary Method; Supplementary Table S1. Initial list of scales and questions considered for MIQ-T; Supplementary Table S2. Exploratory factor analysis with direct oblimin rotation and the factor structure of MIQ-T (Items in Korean); Supplementary Table S3. MIQ-T Short Form (30 Items); Supplementary Table S4. MIQ-T Short Form (15 Items); Supplementary References.
