# Supplementary material for: Development and Validation of the Mood Instability Questionnaire-Trait (MIQ-T)
Source: Medicina (Kaunas). 2021 Aug 18;57(8):838. doi: 10.3390/medicina57080838 (PMC8399587; doi:10.3390/medicina57080838)
Supplement: Supplementary file 1 [file medicina-57-00838-s001.zip › medicina-1268909-supplementary.pdf]

Supplementary Method

Temperament Evaluation of Memphis, Pisa, Paris, and San Diego Autoquestionnaire (TEMPS-A)

TEMPS-A is a measure of an individual's affective temperaments, which are stable traits that influence one's tendency to think or behave in a certain way. There are five temperaments identified in the TEMPS-A: depressive, cyclothymic, hyperthymic, irritable, and anxious. Each temperament dimension includes features of emotional reactivity, psychomotor ability, cognitive activity, circadian rhythm, and social functioning. Both the short version and a modified full version of the self-report questionnaire were utilized in this study. The short version contained 39 items to which the possible response is "Yes" (1) or "No" (0) [1]. The modified TEMPS-A was based on the full version, which had 110 items, and included 20 additional questions that were used in mood disorder clinic at SNUBH and determined to be clinically significant in terms of distinguishing between affective temperaments. For this modified TEMPS-A, the answer choice was altered into a Likert scale ranging from "Never True" (0) to "Very True" (3). A higher score for each respective subscale reflects a higher tendency to behave in that way.

Personality Assessment Inventory for Borderline Features Scale (PAI-BOR)

The Personality Assessment Inventory (PAI) is a self-report questionnaire that assesses personality constructs, such as those relevant for psychopathology and its treatment as well as interpersonal functioning [2]. In the present study, only the Borderline Features Scale (BOR), which is a part of the clinical section of the inventory, was utilized. The PAI-BOR measures one's traits of mood instability, self-harm, problems with identity and difficulties with interpersonal relationships [3]. There are 24 items that are rated on a Likert scale with options ranging from "Never True" (1) to "Very True" (4) [3].

Childhood Trauma Questionnaire, Short Form (CTQ-SF)

CTQ-SF is a retrospective self-report inventory of childhood trauma, including five areas of physical, emotional, and sexual abuse, and physical and emotional neglect [4]. Derived from the original 70 items, the CTQ-SF consists of 28 items with five items in each subscale and three additional items to

ensure validity of the responses. The respondents are given a Likert-type range of answer choices from "Never True" (1) to "Very Often True" (5).

Interpersonal Sensitivity Measure (IPSM)

IPSM is a self-report scale that assesses five factors related to interpersonal relationships: interpersonal awareness, need for approval, separation anxiety, timidity, and fragile inner self [5]. There are 36 items in total, which can be answered in a Likert-type range of options from "Never True" (1) to "Very True" (4). Higher scores reflect that the individual is more sensitive to interpersonal relationships than others are.

Connor-Davidson Resilience Scale (CD-RISC)

Developed by Connor and Davidson [6], CD-RISC is a self-report measure to quantify one's resilience to change and stress. It includes characteristics of one's adaptability and skills related to problem-solving, decision-making, and goal-seeking. There are 25 items in total, which are rated on a 5-point Likert scale from "Not True At All" (0) to "Very True" (4). A higher score indicates that the individual is more resilient to stress and adapts better to change than others.

Behavioral Inhibition/Activation Scale (BIS/BAS* Scale*)

BIS/BAS scale is a self-report questionnaire that measures how one's motivation system works [7]. Behavioral inhibition system (BIS) is perceptive to signs of negative outcomes and restrains behavior. BIS is also sensitive to the negative feelings that may arise due to the adverse experiences, further inhibiting action. On the other hand, behavioral activation system (BAS) is perceptive to signs of reward and encourages behavior. BAS is related to goal-seeking, motivation, and positive feelings [7]. BIS/BAS scale contains 20 items in total, with four subfactors consisting of BIS, BAS Reward Responsiveness, BAS Drive, and BAS Fun Seeking. Originally, the answer choices were written as a Likert scale from "Very True" (1) to "Never True" (4) [7], but for consistency within the present study, the answer choices were reversed, forming a range from "Never True" (1) to "Very True" (4).

Seasonal Pattern Assessment Questionnaire (SPAQ)

SPAQ, developed by Rosenthal, *et al.* [8], encompasses areas of mood variability, changes in energy level and behavior, and changes in food intake and sleep [8]. There are 24 items in total, some of which the respondents are asked to answer with the applicable months, weight change, or number of hours; a few items which should be answered as a "Yes" (1) or "No" (0); and others that have answer choices that are written in a Likert scale from "Does Not Change At All" (0) to "Changes Very Much" (4).

Wender-Utah Rating Scale-25 (WURS-25)

WURS was originally a scale comprising of 61 items, but was shortened into 25 items as these questions showed the greatest score differences between the subjects with attention deficit hyperactivity disorder (ADHD) and those without [9]. WURS-25 is a self-report assessment that retrospectively measures childhood ADHD symptoms and includes factors of attention, irritability, and lack of control [9]. All items are to be answered on a Likert-type range of scores from "Not At All" (0) to "Very Much" (4). A higher score indicates that the individual was more likely to have displayed signs of childhood ADHD.

Beck Anxiety Inventory (BAI)

Developed by Beck, *et al.* [10], BAI is a self-report questionnaire encompassing areas of physical and psychological symptoms related to anxiety. Some examples of physical symptoms that are asked include feeling dizzy, heart pounding, and difficulty breathing, while psychological symptoms include feeling nervous and fear of losing control. BAI consists of 21 items and are rated on a Likert scale ranging from "Not At All" (0) to "Severely" (3), based on how much the individual has been bothered by each symptom over the past seven days.

Zung Self-Rating Depression Scale (SDS)

SDS, developed by Zung [11], is a 20-item diagnostic inventory that measures an individual's depression severity. It includes factors of mood, cognition, and behavior, including reactions to stress such as appetite and sleep. The respondents are expected to reflect on how they felt and behaved during the past

two weeks while answering the questions. The items are scored on a Likert scale from 1 to 4, but the statements that correspond to the numeral values depend on the valence of the statement. For negative statements, such as “I feel down-hearted and blue,” the response “A Little of the Time” has a value of 1 and “Most of the Time” has a value of 4, while for positive statements, such as “Morning is when I feel best”, the response “A Little of the Time” has a value of 4 and “Most of the Time” has a value of 1 [11].

Table S1. Initial list of scales and questions considered for MIQ-T.

Name of Scale	Item Description (English)	Item Description (Korean)
Temperament Evaluation of Memphis, Pisa, Paris, and San Diego Autoquestionnaire (TEMPS-A) – Modified Version	My ability to think varies greatly from sharp to dull for no apparent reason.	분명한 이유 없이 사고력이 날카롭다가 둔했다가 한다.
	I constantly switch from being lively and sluggish.	계속해서 활기찼다가 침울했다가 한다.
	I get sudden shifts in mood and energy.	기분과 에너지의 변화가 심하게 오락가락 한다.
	The way I see things is sometimes vivid, but at other times lifeless.	때때로 세상이 활기 있게 보이기도 하고 무기력하게 보이기도 한다.
	My mood often changes for no reason.	이유 없이 기분이 변하곤 한다.
	I go back and forth between being outgoing and being withdrawn from others.	나는 사람들과의 만남에서 적극적이었다가 소극적이었다가 한다.
	My moods and energy are either high or low, rarely in between.	내 기분과 에너지 상태는 높거나 낮은 상태이며 그 중간일 때는 거의 없다.
	I go back and forth between feeling overconfident and feeling unsure of myself	나는 지나치게 자신감이 넘치거나 자신이 없거나 한다.
	My need for sleep varies a lot from just a few hours to more than 9 hours.	몇 시간밖에 못 자도 괜찮다가 9 시간 이상 자도 잠이 부족할 때가 있다.
	I sometimes go to bed feeling great and wake up in the morning feeling like life is not worth living.	기분 좋게 자고 아침에 일어났는데 인생은 살 가치가 없다고 생각할 때가 있다.
	I am the kind of person who can be sad and happy at the same time.	나는 슬픔과 행복을 동시에 느낄 수 있는 사람이다.
	I often feel tired for no reason.	나는 이유없이 지칠 때가 자주 있다
	I can really like someone a lot, and then completely lose my interest in them.	나는 어떤 사람을 매우 좋아하다가도 아예 관심없어지기도 한다
	I often blow up at people and then feel guilty about it.	나는 자주 사람들에게 화를 폭발하고나서 죄책감을 느낀다.

	I often start things and the lose interest before finishing them.	나는 종종 일을 시작하고 끝내기 전에 관심을 잃는다.
	I sometimes go to bed feeling down and wake up in the morning feeling terrific.	가끔 나는 우울하게 잠이 들었다가 아침에 일어나서는 기분이 좋아진다.
	I am told that I often get pessimistic about things and forget previous happy times.	나는 매우 비관적이 되어 행복했던 일들을 생각하지 못한다.
	I feel my emotions intensely.	나는 감정을 강하게 느낀다.
	I daydream a great deal about things that other people consider impossible to achieve.	나는 다른 사람들이 불가능하다고 느끼는 것에 대해 공상할 때가 많다.
	I often have a strong urge to do outrageous things.	나는 종종 잔인한 행동을 하고 싶은 충동이 들 때가 있다.
	I am an insecure person.	나는 불안정한 사람이다.
	I get stressed by minor changes in my daily life.	나는 일상의 사소한 변화에도 스트레스를 받는다.
	When I experience something difficult, I get depressed easily.[+]	나는 힘든 일을 겪으면 기분이 쉽게 우울해진다.[+]
	When I experience something difficult, my moods become unstable.[+]	나는 힘든 일을 겪으면 감정 기복이 심해진다.[+]
	When I experience something difficult, I am more likely to get irritable.[+]	나는 어려운 일을 겪으면 짜증이 늘어난다.[+]
	When I experience something difficult, I feel anxious.[+]	나는 힘든 일을 겪을 때, 불안 초조하다.[+]
	When something good happens, I feel despondent.[+]	나는 좋은 일이 있을 때, 괜히 의기소침해진다.[+]
	When something good happens, my moods becomes unstable.[+]	나는 좋은 일을 겪으면 기분이 들쑥날쑥해진다.[+]
	When something good happens, I feel intensely euphoric.[+]	나는 좋은 일을 겪으면 과도하게 즐거워한다.[+]
	When something good happens, I feel irritable.[+]	나는 좋은 일이 있을 때, 오히려 예민해진다.[+]
	When something good happens, I feel anxious.[+]	나는 좋은 일이 있을 때, 막연히 불안해진다.[+]

	When I am busy, I feel depressed.[+]	나는 바쁜 상황이 되면 기분이 우울해진다.[+]
	When I am busy, my moods become unstable.[+]	나는 바쁜 상황이 되면 기분이 들쑥날쑥해진다.[+]
	When I am busy, I feel more energetic.[+]	나는 바쁜 상황에서 더 활력적이게 된다.[+]
	When I am busy, I easily get irritable.[+]	나는 바쁜 상황에서 쉽게 예민해진다.[+]
	When I am busy, I feel anxious.[+]	나는 바쁜 상황이 되면 불안해진다.[+]
	When I have a lot of free time, I feel depressed.[+]	나는 한가로울 때 기분이 울적하다.[+]
	When I have a lot of free time, my moods become unstable.[+]	나는 한가로운 상황에서 기분이 들쑥날쑥해진다.[+]
	When I have a lot of free time, I feel irritable.[+]	나는 한가로운 상황에서 짜증을 느낀다.[+]
	When I have a lot of free time, I feel anxious.[+]	나는 한가로울 때, 막연히 불안해진다.[+]
Connor-Davison Resilience Scale (CD-RISC)	I am able to adapt to change.*	변화가 일어날 때 적응할 수 있다.*
	Even when I am under pressure, I can focus and think clearly.*	스트레스를 받을 때에도, 나는 집중력과 사고력을 잘 유지한다.*
	I can handle unpleasant and painful feelings, such as sadness, fear, and anger.*	슬픔, 공포, 그리고 분노와 같은 불쾌하거나 고통스러운 감정들을 잘 처리할 수 있다.*
Behavioral Inhibition/Activation Scale (BIS/BAS)	When I get something I want, I feel excited and energized.	내가 원하는 어떤 것을 얻게 되면, 나는 흔히 흥분하고 기운이 넘친다.
	Criticism or scolding hurts me quite a bit.	비판을 받거나 꾸중을 듣게 되면 나는 마음이 많이 상한다.
	I feel pretty worried or upset when I think or know somebody is angry at me.	누군가가 나에게 화를 내고 있다는 것을 알게 되면, 나는 많이 근심하거나 마음 심란해 한다.
	When I see an opportunity for something I like, I get excited right away.	내가 좋아하는 어떤 것을 볼 기회를 가지게 되면 나는 곧바로 흥분한다.
	I often act on the spur of the moment.	나는 보통 ‘기분파’라는 말을 자주 듣는 편이다.
	If I think something unpleasant is going to happen, I usually get pretty "worked up".	나쁜 일이 일어날지도 모르겠다는 생각이 들면, 나는 보통 크게 흥분하게 된다.

	When good things happen to me, it affects me strongly.	내게 좋은 일이 일어난다면, 나는 보통 그 일로 인해 크게 영향을 받곤 한다.
	It would excite me to win a contest.	나는 경기에서 이기면 보통 매우 흥분한다.
Seasonal Pattern Assessment Questionnaire (SPAQ)	How much do you experience seasonal variation in social activity?	사회적 활동의 정도가 계절에 따라 얼마나 변합니까?
	How much do you experience seasonal variation in mood?	기분상태가 계절에 따라 얼마나 변합니까?
	How much do you experience seasonal variation in energy?	활력정도가 계절에 따라 얼마나 변합니까?
Personality Assessment Inventory - Borderline Features Scale (PAI-BOR)	My mood shifts very suddenly.	나는 기분이 매우 갑작스럽게 변한다.
	My attitudes about myself changes a lot.	나 자신에 대한 태도가 크게 변화한다.
	My relationships have been stormy.	나의 대인관계는 매우 불안정했다.
	My moods can become very intense.	내 기분은 아주 강렬해지곤 한다.
	Sometimes, I feel terribly empty inside.	때때로 나는 몹시 공허한 느낌이 든다.
	My mood is very steady.*	내 기분은 매우 안정되어 있다.*
Wender-Utah Rating Scale-25 (WURS-25)	As a child, I had temper outbursts and tantrums.	어렸을 때 나는 분노발작, 감정 폭발이 있었다.
	As a child, I was hot- or short-tempered, with a low boiling point.	어렸을 때 나는 쉽게 자극되어 흥분하였다.
	As a child, I was moody with ups and downs.	어렸을 때 나는 변덕스럽고 기분에 기복이 있었다.
	As a child, I acted without thinking and I was impulsive.	어렸을 때 나는 생각없이 행동해버리고 충동적이었다.
	As a child, I lost control of myself.	어렸을 때 나는 자신을 통제하지 못하였다.

Notes: + indicates items developed in SNUBH

* indicates items that were reverse-coded.

Table S2. Exploratory factor analysis with direct oblimin rotation and the factor structure of MIQ-T (Items in Korean).

Item (Korean)	Factor 1	Factor 2	Factor 3	Factor 4	Factor 5
Factor #1 – Lability					
나는 한가로운 상황에서 기분이 들쑥날쑥해진다.	**.759**	.123	-.047	-.030	-.024
나는 한가로울 때 기분이 울적하다.	**.722**	-.003	.026	-.076	.048
나는 한가로운 상황에서 짜증을 느낀다.	**.695**	-.103	.039	-.113	.125
계속해서 활기찼다가 침울했다가 한다.	**.669**	.164	.156	.047	-.180
나는 좋은 일이 있을 때, 오히려 예민해진다.	**.666**	-.160	-.053	.015	.345
기분과 에너지의 변화가 심하게 오락가락 한다.	**.641**	.204	.165	.048	-.249
나는 한가로울 때, 막연히 불안해진다.	**.634**	-.173	.145	-.014	.177
나는 좋은 일이 있을 때, 괜히 의기소침해진다.	**.630**	-.289	-.031	-.066	.320
때때로 세상이 활기 있게 보이기도 하고 무기력하게 보이기도 한다.	**.628**	.181	.121	.010	-.088
분명한 이유 없이 사고력이 날카롭다가 둔했다가 한다.	**.611**	.039	.138	.042	-.092
몇 시간밖에 못 자도 괜찮다가 9 시간 이상 자도 잠이 부족할 때가 있다.	**.593**	.108	-.016	.081	-.086
나는 좋은 일을 겪으면 기분이 들쑥날쑥해진다.	**.590**	-.013	.020	.137	.184
이유 없이 기분이 변하곤 한다.	**.588**	.059	.230	.042	-.156
나는 어떤 사람을 매우 좋아하다가도 아예 관심없어지기도 한다.	**.582**	.141	.035	.045	-.047
내 기분과 에너지 상태는 높거나 낮은 상태이며 그 중간일 때는 거의 없다.	**.570**	.054	.149	.159	-.236
나는 사람들과의 만남에서 적극적이었다가 소극적이었다가 한다.	**.540**	.147	.063	.128	-.065
좋은 일이 있을 때, 막연히 불안해진다.	**.519**	-.365	.172	.109	.335
나 자신에 대한 태도가 크게 변화한다.	**.518**	.268	.094	.149	-.116
기분 좋게 자고 아침에 일어났는데 인생은 살 가치가 없다고 생각할 때가 있다.	**.503**	-.266	.291	.121	-.148
나는 기분이 매우 갑작스럽게 변한다.	**.489**	.232	.121	.202	-.290
때때로 나는 몹시 공허한 느낌이 든다.	**.484**	-.022	.270	.201	-.081
나는 다른 사람들이 불가능하다고 느끼는 것에 대해 공상할 때가 많다.	**.479**	.158	-.025	.159	.081

나는 지나치게 자신감이 넘치거나 자신이 없거나 한다.	**.448**	.121	.167	.203	-.123
나는 이유없이 지칠 때가 자주 있다.	**.448**	-.081	.341	.115	-.022
나는 바쁜 상황에서 더 활력적이 된다.	**.413**	.317	-.372	-.058	-.033
나는 슬픔과 행복을 동시에 느낄 수 있는 사람이다.	**.402**	.164	-.062	.057	.042
Factor #2 – Upward Tendency					
내가 원하는 어떤 것을 얻게 되면, 나는 흔히 흥분하고 기운이 넘친다.	-.041	**.742**	.073	.023	.058
내게 좋은 일이 일어난다면, 나는 보통 그 일로 인해 크게 영향을 받곤 한다.	.037	**.696**	.112	-.034	.068
내가 좋아하는 어떤 것을 볼 기회를 가지게 되면 나는 곧바로 흥분한다.	-.043	**.669**	.085	.204	.140
나는 경기에서 이기면 보통 매우 흥분한다.	.019	**.575**	-.031	.052	.098
나는 보통 '기분파' 라는 말을 자주 듣는 편이다.	.135	**.556**	.001	.257	.026
나는 좋은 일을 겪으면 과도하게 즐거워한다.	.207	**.530**	.040	.051	-.012
Factor #3 – Downward Tendency					
나는 힘든 일을 겪을 때, 불안 초조하다.	.106	.102	**.671**	-.006	.011
나는 힘든 일을 겪으면 기분이 쉽게 우울해진다.	.157	.044	**.670**	.039	-.043
비판을 받거나 꾸중을 듣게 되면 나는 마음이 많이 상한다.	-.096	.360	**.616**	-.138	.062
나는 바쁜 상황이 되면 불안해진다.	.044	-.085	**.608**	.064	.352
누군가가 나에게 화를 내고 있다는 것을 알게 되면, 나는 많이 근심하거나 마음 심란해 한다.	-.052	.374	**.593**	-.209	.051
나는 어려운 일을 겪으면 짜증이 늘어난다.	.047	.103	**.581**	.174	.052
변화가 일어날 때 적응할 수 있다. *	.060	-.138	**.557**	-.044	-.179
슬픔, 공포, 그리고 분노와 같은 불쾌하거나 고통스러운 감정들을 잘 처리할 수 있다. *	.155	-.041	**.550**	.049	-.147
나는 일상의 사소한 변화에도 스트레스를 받는다.	.336	-.020	**.534**	-.007	.031
나는 힘든 일을 겪으면 감정 기복이 심해진다.	.328	.159	**.532**	.005	-.018
나는 바쁜 상황이 되면 기분이 우울해진다.	.035	-.129	**.526**	.024	.389

스트레스를 받을 때에도, 나는 집중력과 사고력을 잘 유지한다. *	.101	-.078	**.524**	.199	-.234
나는 바쁜 상황에서 쉽게 예민해진다.	.032	.037	**.515**	.184	.303
나는 불안정한 사람이다.	.397	-.130	**.491**	.154	-.123
나는 매우 비관적이 되어 행복했던 일들을 생각하지 못한다.	.220	-.173	**.485**	.247	-.025
내 기분은 매우 안정되어 있다. *	.320	-.110	**.448**	.105	-.258
나쁜 일이 일어날지도 모르겠다는 생각이 들면, 나는 보통 크게 흥분하게 된다.	.112	.378	**.435**	-.052	.130
나는 바쁜 상황이 되면 기분이 들쑥날쑥해진다.	.231	.096	**.419**	.105	.312
Factor #4 – Childhood Instability					
어렸을 때 나는 쉽게 자극되어 흥분하였다.	-.134	.057	-.036	**.862**	.071
어렸을 때 나는 생각없이 행동해버리고 충동적이었다.	-.099	.053	-.088	**.835**	.014
어렸을 때 나는 변덕스럽고 기분에 기복이 있었다.	.035	.021	-.060	**.822**	.121
어렸을 때 나는 자신을 통제하지 못하였다.	.006	-.028	-.081	**.765**	.109
어렸을 때 나는 분노발작, 감정 폭발이 있었다.	-.025	-.005	-.011	**.705**	-.010
나의 대인관계는 매우 불안정했다.	.099	-.091	.353	**.418**	-.160
Factor #5 – Seasonality					
활력 정도가 계절에 따라 얼마나 변합니까?	.023	.236	-.025	.151	**.555**
기분상태가 계절에 따라 얼마나 변합니까?	.061	.285	.019	.122	**.523**
사회적 활동의 정도가 계절에 따라 얼마나 변합니까?	-.015	.215	.071	.136	**.522**

Notes: Factor loadings with absolute value greater than .40 are shown in bold. * indicates items that were reverse-coded. Extraction Method: Principle Component Analysis. Extraction Method: Oblimin with Kaiser Normalization.

Table S3. MIQ-T Short Form (30 Items).

Item (English)	Item (Korean)	Factor loading			
		Factor 1	Factor 2	Factor 3	Factor 4
Factor #1 – Lability					
My ability to think varies greatly from sharp to dull for no apparent reason.	분명한 이유 없이 사고력이 날카롭다가 둔했다가 한다.	**.617**	.041	.143	.049
I constantly switch from being lively and sluggish.	계속해서 활기찼다가 침울했다가 한다.	**.696**	.149	.164	.052
I get sudden shifts in mood and energy.	기분과 에너지의 변화가 심하게 오락가락 한다.	**.680**	.189	.170	.052
The way I see things is sometimes vivid, but at other times lifeless.	때때로 세상이 활기 있게 보이기도 하고 무기력하게 보이기도 한다.	**.650**	.158	.122	.016
My mood often changes for no reason.	이유 없이 기분이 변하곤 한다.	**.607**	.045	.235	.041
I go back and forth between being outgoing and being withdrawn from others.	나는 사람들과의 만남에서 적극적이었다가 소극적이었다가 한다.	**.560**	.137	.064	.135
My moods and energy are either high or low, rarely in between.	내 기분과 에너지 상태는 높거나 낮은 상태이며 그 중간일 때는 거의 없다.	**.593**	.048	.167	.160
My need for sleep varies a lot from just a few hours to more than 9 hours.	몇 시간밖에 못 자도 괜찮다가 9 시간 이상 자도 잠이 부족할 때가 있다.	**.616**	.102	-.012	.084
I can really like someone a lot, and then completely lose my interest in them.	나는 어떤 사람을 매우 좋아하다가도 아예 관심없어지기도 한다.	**.592**	.118	.045	.044
When something good happens, my moods becomes unstable.	나는 좋은 일을 겪으면 기분이 들쑥날쑥해진다.	**.576**	-.030	-.019	-.080
When something good happens, I feel anxious.	나는 좋은 일이 있을 때, 막연히 불안해진다.	**.552**	-.367	.189	.102

When I have a lot of free time, I feel depressed.	나는 한가로울 때 기분이 울적하다.	**.718**	-.033	.022	-.068
When I have a lot of free time, my moods become unstable.	나는 한가로운 상황에서 기분이 들쑥날쑥해진다.	**.775**	0.74	-0.50	-.021
My attitudes about myself changes a lot.	나 자신에 대한 태도가 크게 변화한다.	**.553**	.240	.100	.148
Factor #2 – Upward Tendency					
When I get something I want, I feel excited and energized.	내가 원하는 어떤 것을 얻게 되면, 나는 흔히 흥분하고 기운이 넘친다.	.019	**.735**	.050	.023
When I see an opportunity for something I like, I get excited right away	내가 좋아하는 어떤 것을 볼 기회를 가지게 되면 나는 곧바로 흥분한다.	.015	**.674**	.064	.202
I often act on the spur of the moment.	나는 보통 '기분파' 라는 말을 자주 듣는 편이다.	.190	**.529**	-.016	.256
When good things happen to me, it affects me strongly.	내게 좋은 일이 일어난다면, 나는 보통 그 일로 인해 크게 영향을 받곤 한다.	.092	**.692**	.083	-.022
It would excite me to win a contest.	나는 경기에서 이기면 보통 매우 흥분한다.	.059	**.572**	-.049	.055
Factor #3 – Downward Tendency					
I get stressed by minor changes in my daily life.	나는 일상의 사소한 변화에도 스트레스를 받는다.	.203	-.019	**.533**	-.010
When I experience something difficult, I get depressed easily.	나는 힘든 일을 겪으면 기분이 쉽게 우울해진다.	.163	.056	**.671**	.044
When I experience something difficult, I am more likely to get irritable.	나는 어려운 일을 겪으면 짜증이 늘어난다.	.048	.116	**.580**	.169
When I experience something difficult, I feel anxious.	나는 힘든 일을 겪을 때, 불안 초조하다.	.108	.101	**.670**	.000
Even when I am under pressure, I can focus and think clearly. *	스트레스를 받을 때에도, 나는 집중력과 사고력을 잘 유지한다. *	.108	-.059	**.549**	.193

I can handle unpleasant and painful feelings, such as sadness, fear, and anger. *	슬픔, 공포, 그리고 분노와 같은 불쾌하거나 고통스러운 감정들을 잘 처리할 수 있다. *	.170	-.036	**.561**	.045
Factor #4 – Childhood Instability					
As a child, I had temper outbursts and tantrums.	어렸을 때 나는 분노발작, 감정 폭발이 있었다.	.004	.001	-.002	**.711**
As a child, I was hot- or short-tempered, with a low boiling point.	어렸을 때 나는 쉽게 자극되어 흥분하였다.	-.114	.047	-.023	**.856**
As a child, I was moody with ups and downs.	어렸을 때 나는 변덕스럽고 기분에 기복이 있었다.	.048	.007	-.052	**.820**
As a child, I acted without thinking and I was impulsive.	어렸을 때 나는 생각없이 행동해버리고 충동적이었다.	-.079	.044	-.074	**.826**
As a child, I lost control of myself.	어렸을 때 나는 자신을 통제하지 못하였다.	.010	-.039	-.061	**.765**

Notes: The 30-item MIQ-T Short Form (MIQ-T SF-30) was created by a combination of two methods. First, a new exploratory factor analysis was conducted with the items of the MIQ-T full questionnaire, and only items that had factor loadings greater than 0.50 on its original factor and low loadings (less than 0.30) on other factors were retained. Second, of the remaining items, item-total correlations were computed for each factor and items that had a correlation coefficient greater than 0.50 were kept. * indicates items that were reverse-coded.

Table S4. MIQ-T Short Form (15 Items).

Item (English)	*Item (Korean)*	*Factor 1*	*Factor 2*	*Factor 3*	*Factor 4*
Factor #1 – Lability					
My ability to think varies greatly from sharp to dull for no apparent reason.	분명한 이유 없이 사고력이 날카롭다가 둔했다가 한다.	**.769**	-.065	.031	-.039
I constantly switch from being lively and sluggish.	계속해서 활기찼다가 침울했다가 한다.	**.875**	.031	.029	-.066
I get sudden shifts in mood and energy.	기분과 에너지의 변화가 심하게 오락가락 한다.	**.885**	.051	.014	-.079
The way I see things is sometimes vivid, but at other times lifeless.	때때로 세상이 활기 있게 보이기도 하고 무기력하게 보이기도 한다.	**.755**	-.051	.043	-.044
My moods and energy are either high or low, rarely in between.	내 기분과 에너지 상태는 높거나 낮은 상태이며 그 중간일 때는 거의 없다.	**.773**	-.070	-.004	.063
When I have a lot of free time, my moods become unstable.	나는 한가로운 상황에서 기분이 들쑥날쑥해진다.	**.737**	.031	-.048	.013
Factor #2 – Upward Tendency					
When I get something I want, I feel excited and energized.	내가 원하는 어떤 것을 얻게 되면, 나는 흔히 흥분하고 기운이 넘친다.	.012	**.811**	.060	-.047
When I see an opportunity for something I like, I get excited right away	내가 좋아하는 어떤 것을 볼 기회를 가지게 되면 나는 곧바로 흥분한다.	.080	**.757**	.015	.135
When good things happen to me, it affects me strongly.	내게 좋은 일이 일어난다면, 나는 보통 그 일로 인해 크게 영향을 받곤 한다.	.079	**.724**	.090	-.044
Factor #3 – Downward Tendency					
When I experience something difficult, I get depressed easily.	나는 힘든 일을 겪으면 기분이 쉽게 우울해진다.	.020	.054	**.818**	-.025

When I experience something difficult, I am more likely to get irritable.	나는 어려운 일을 겪으면 짜증이 늘어난다.	-.068	.144	**.735**	.078
When I experience something difficult, I feel anxious.	나는 힘든 일을 겪을 때, 불안 초조하다.	-.086	.095	**.849**	-.028
Factor #4 – Childhood Instability					
As a child, I was hot- or short-tempered, with a low boiling point.	어렸을 때 나는 쉽게 자극되어 흥분하였다.	-.110	.089	.030	**.861**
As a child, I was moody with ups and downs.	어렸을 때 나는 변덕스럽고 기분에 기복이 있었다.	.087	.016	-.009	**.820**
As a child, I acted without thinking and I was impulsive.	어렸을 때 나는 생각없이 행동해버리고 충동적이었다.	-.041	.046	-.041	**.817**

Notes: The 15-item MIQ-T Short Form was developed via conducting a new exploratory factor analysis on the MIQ-T SF-30 and retaining items with the highest factor loadings. For Factor #1, the longest factor, 6 items with the highest factor loadings were kept, while for the other 3 factors, 3 items were retained for each. * indicates items that were reverse-coded.

Supplementary References

1. Akiskal, H.S.; Mendlowicz, M.V.; Jean-Louis, G.; Rapaport, M.H.; Kelsoe, J.R.; Gillin, J.C.; Smith, T.L. TEMPS-A: validation of a short version of a self-rated instrument designed to measure variations in temperament. *J Affect Disord* **2005**, *85*, 45-52, doi:10.1016/j.jad.2003.10.012.
2. Morey, L.C. *The personality assessment inventory: Professional manual*; Psychological Assessment Resources: Odessa, FL, 1991.
3. Morey, L.C.; Ambwani, S. The personality assessment inventory. In *The SAGE handbook of personality theory and assessment*, Boyle, G.J., Matthews, G., Saklofske, D.H., Eds. SAGE Publications: New York, NY, 2008; Vol. 2, pp. 626-645.
4. Bernstein, D.P.; Stein, J.A.; Newcomb, M.D.; Walker, E.; Pogge, D.; Ahluvalia, T.; Stokes, J.; Handelsman, L.; Medrano, M.; Desmond, D., et al. Development and validation of a brief screening version of the Childhood Trauma Questionnaire. *Child Abuse Negl* **2003**, *27*, 169-190, doi:10.1016/s0145-2134(02)00541-0.
5. Boyce, P.; Parker, G. Development of a scale to measure interpersonal sensitivity. *Aust N Z J Psychiatry* **1989**, *23*, 341-351.
6. Connor, K.M.; Davidson, J.R. Development of a new resilience scale: the Connor-Davidson Resilience Scale (CD-RISC). *Depress Anxiety* **2003**, *18*, 76-82, doi:10.1002/da.10113.
7. Carver, C.S.; White, T.L. Behavioral inhibition, behavioral activation, and affectiv responses to impending reward and punishment: The BIS/BAS Scales. *J Pers Soc Psychol* **1994**, *67*, 319-333.
8. Rosenthal, N.E.; Genhart, M.; Sack, D.A.; Skwerer, R.G.; Wehr, T.A. Seasonal affective disorder: Relevance for treatment and research of bulimia. In *Psychobiology of Bulimia*, Hudson, E.L., Pope, H.G., Eds. American Psychiatric Press: Washington, D.C., 1987.
9. Ward, M.F.; Wender, P.H.; Reimherr, F.W. The Wender Utah Rating Scale: an aid in the retrospective diagnosis of childhood attention deficit hyperactivity disorder. *Am J Psychiatry* **1993**, *150*, 885-890, doi:10.1176/ajp.150.6.885.
10. Beck, A.T.; Epstein, N.; Brown, G.; Steer, R.A. An inventory for measuring clinical anxiety: psychometric properties. *J Consult Clin Psychol* **1988**, *56*, 893-897, doi:10.1037//0022-006x.56.6.893.
11. Zung, W.W.K. A Self-Rating Depression Scale. *Arch Gen Psychiatry* **1965**, *12*, 63-70, doi:10.1001/archpsyc.1965.01720310065008.
